# Vernal keratoconjunctivitis in twins: case report and literature review

**DOI:** 10.1186/s13052-021-01073-w

**Published:** 2021-06-12

**Authors:** Maria Cristina Artesani, Mariacristina Esposito, Maurizio Mennini, Marco Andreani, Franco Locatelli, Luca Buzzonetti, Alessandro Fiocchi

**Affiliations:** 1 Translational Specialized Pediatrics Research Area, Allergic Diseases Research Unit, Bambino Gesù Children’s Hospital, IRCCS, Piazza San’Onofrio, 4, 00165 Rome, Italy; 2 Ophthalmology Department, Bambino Gesù Children’s Hospital, IRCCS, Rome, Italy; 3 Laboratory of Immunogenetics of Transplant, Department of Pediatric Hematology/Oncology and of Cell and Gene Therapy, Bambino Gesù Children’s Hospital, IRCCS, Rome, Italy; 4 Department of Pediatric Hematology/Oncology and of Cell and Gene Therapy, Bambino Gesù Children’s Hospital, IRCCS, Rome, Italy

Vernal keratoconjunctivitis (VKC) is a chronic bilateral seasonal allergic inflammatory disease with a prevalence of < 1 case out of 10,000 in Europe [1], which occurs mainly in pediatric age. The diagnosis is generally confirmed by the finding at the ocular examination of conjunctival hyperemia, papillary hypertrophy in the tarsal conjunctiva, giant papillae and Trantas dots in the limbus region.

Few studies evaluated the association of specific HLA genes with VKC. In an Italian pediatric study, HLA class I A32 was found more frequent in familiar than sporadic forms of VKC [2]. In another pediatric population, patients with VKC presented more frequently HLA-DRB1*01 and DRB1*16, while the DRB1*13 was negatively associated with VKC. The DRB1*01 and DRB1*16 families of alleles are in strong linkage disequilibrium (LD) with the DQB1*05 allele, that was found significantly more frequent in VKC patients than in controls [3]. In this context, HLA analysis of monozygotic twin patients with VKC may provide useful information to clarify the haplotypes potentially implicated in the pathogenesis of the disease. Furthermore, differently from previous reported data, in our investigation we applied a next generation sequencing (NGS) typing approach to determine the different HLA alleles of class I and II present in the studied patients.

After obtaining the informed consent from patients and their parents and the approval of our local Ethics Committee, we describe here the assessment of HLA in a couple of monozygotic twins and in their father, all with VKC. Two 10-years-old Caucasian male monozygotic twins with history of mild intermittent allergic rhinoconjuctivitis to dust mites, as determined by positive skin prick testing, came to our observation due to the appearance of bilateral conjunctivitis in spring-summer time which responded only to steroid topic therapy. The patients complained of ocular itching, burning, watering and mucoid stringy discharge and intense photophobia. On slit-lamp examination, the children showed conjunctival hyperemia, papillary hypertrophy, giant papillae and Tranta’s nodules (Fig. 1 a, b). Vernal keratoconjunctivitis was diagnosed and the disease activity was graded, according to the Bonini VKC severity score [4], as severe (grade 3) for both twins. A successful topical immunosuppressant therapy with cyclosporin 1% was initiated. Their father was diagnosed with VKC, while their mother had no ocular symptoms or signs. We performed the HLA typing at high resolution of the DNA of the two patients and of all the family members available, by NGS.

**Fig. 1:**
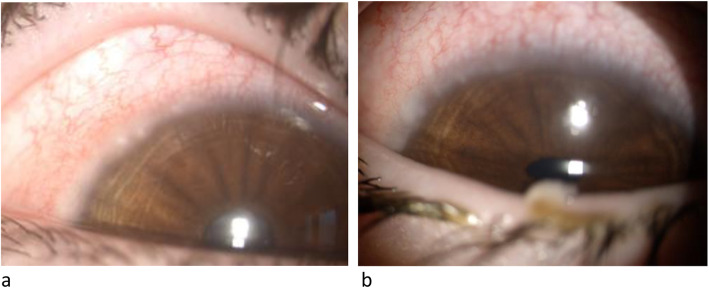
Slit-lamp examination, twin n.1 (a) and twin n.2 (b)

DNA samples were extracted using an EZ1 DSP DNA Blood kit (Qiagen - Thermo Fisher Scientific Walthman, Massacchussets, USA) on an automatic EZ1 Advanced XL instrument (Qiagen- Thermo Fisher Scientific Walthman, Massacchussets, USA) from peripheral blood samples. HLA genotyping was obtained after a library preparation, using the AllType kit (One Lambda, Canoga Park, California) and run on the Ion Torrent S5 XL platform (Thermo Fisher Scientific Walthman, Massacchussets, USA). These kits use a single multiplexed polymerase chain reaction (PCR) to amplify the full HLA-A/B/C/DQA1/DPA1 gene sequences and from exon 2 to the 30UTR of the HLADRB1/3/4/5/DQB1/DPB1 genes. Reads were analyzed using the HLA TypeStream Visual Software (TSV) (One Lambda), ver. 1.1.0.27232.

The twins’ haplotype is presented in detail in Table 1, reporting the different whole HLA haplotypes of all the different family members investigated. The patients presented three different HLA alleles reported to be strongly associated with the developing of VKC: DQB1*05:01:01 and HLA-DRB1*01:01:01 alleles, inherited from the father, also affected by VKC, and the HLA-A*32:01:01 allele, present in the mother haplotype (Fig. 2). The mother presents also the HLA-DRB1*13:02:01 allele, considered protective for VKC [2].

**Table 1:** HLA typing, at high resolution of the DNA of the two patients and of all the family members, by next generation sequencing (NGS) by haplotype segregation

Family Relationship	Haplotype	HLA-A*	HLA-B*	HLA-C*	HLA-DRB1*	HLA-DQB1*	HLA-DRB3*	HLA-DQA1*	HLA-DPB1*
Father	a	03:01:01	35:01:01	04:01:01	01:01:01	05:01	–	01:01:01	04:02P
Father	b	03:01:01	35:01:01	07:04:01	11:01:01	03:01P	02:02:01	05:05:01	04:01P
Mother	c	32:01:01	15:17:01	07:01:02	13:02:01	06:04:01	03:01:01	01:02:01	04:01P
Mother	d	01:01:01	18:01:01	12:03:01	11:04:01	03:01P	02:02:01	05:05:01	14:01P
Patient 1	a	03:01:01	35:01:01	04:01:01	01:01:01	05:01	–	01:01:01	04:02P
Patient 1	c	32:01:01	15:17:01	07:01:02	13:02:01	06:04:01	03:01:01	01:02:01	04:01P
Patient 2	a	03:01:01	35:01:01	04:01:01	01:01:01	05:01	–	01:01:01	04:02P
Patient 2	c	32:01:01	15:17:01	07:01:02	13:02:01	06:04:01	03:01:01	01:02:01	04:01P

**Fig. 2:**
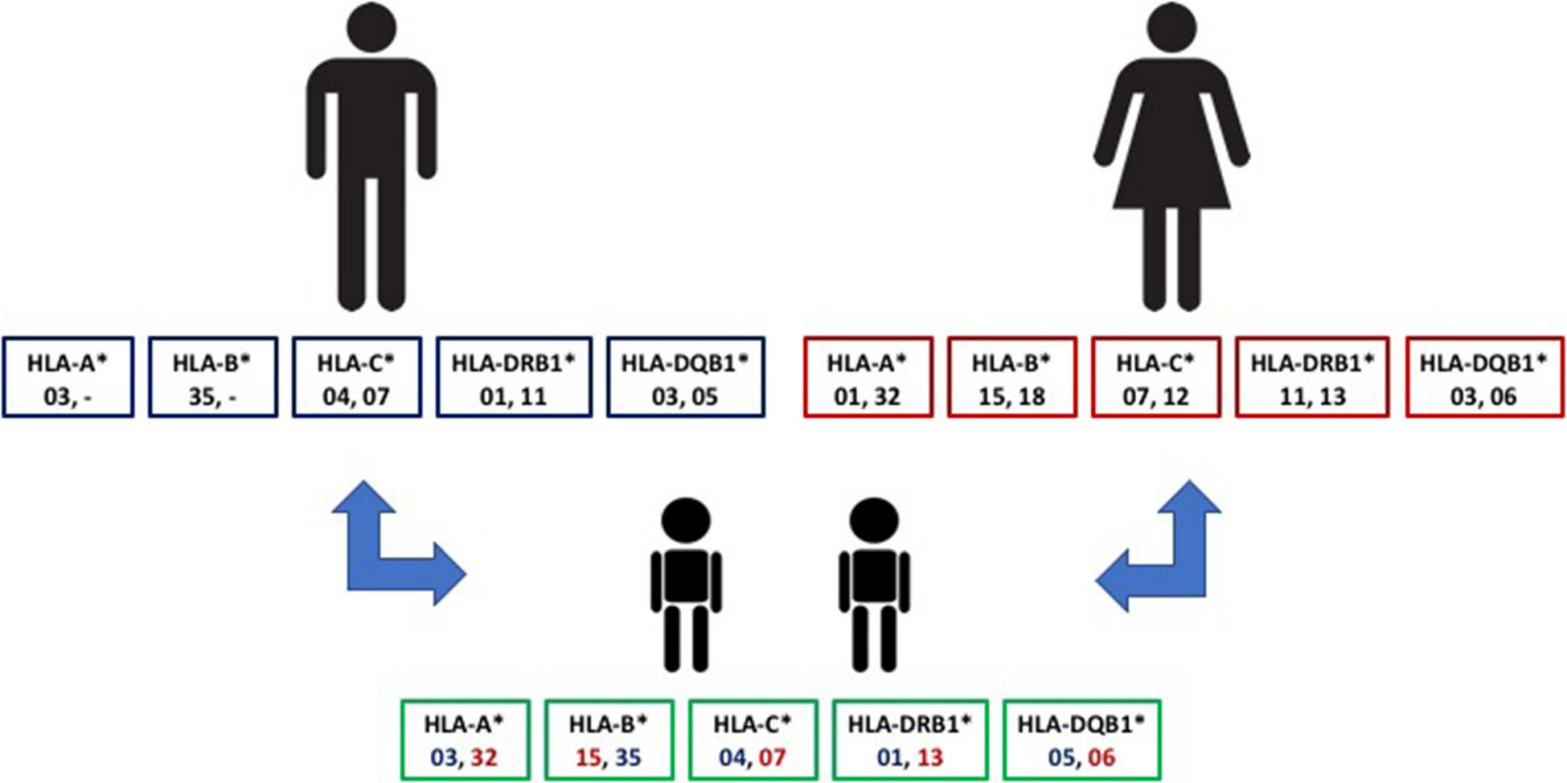
Familiar HLA typing

As reported by Zicari et al., DQB1*05 has been already associated to VKC, both in presence of DRB1*01 and DRB1*16 families of alleles typed at low resolution [3]. It is well known from the literature that these two families of alleles are in strong LD with DQB1*05:01 and DQB1*05:02, respectively, specified as DRB1*01:01-DQB1*05:01 and DRB1*16:01-DQB1*05:02.

VKC is an immune-based disorder, most likely with a genetic predisposition [5], but many questions about its pathogenesis remain still unanswered [6]. Further studies need to be carried out to elucidate the full spectrum of immune-genetics of VKC in order to obtain a real stratification of risk. At the moment, our case report suggests that the presence of VKC cases in families should be valorized and approached as a potentially genetically determined condition.

To identify other similar cases, a search of Medline via PubMed and Google Scholar was conducted using the following search strings: “keratoconjunctivis” or “vernal” or “vernal keratoconjunctivis” AND “twins”. The search was restricted to scientific literature published up to January 2021. We identified four reports of VKC in twins, but three of them did not evaluated the relevance of HLA assessment [7–9].

So only one case report described the assessment of HLA haplotypes A2, A11, B27, B61, DR1, and DR4 in a couple of twins affected by atopic dermatitis, allergic rhino-conjunctivitis and food allergy, but it is not possible to deduce from the text whether the described conjunctivitis could be a VKC or just an allergic conjunctivitis [10].

So far, at our knowledge, this is the first description of HLA haplotypes through NGS approach in twins with VKC.

## Data Availability

The datasets generated during and/or analysed during the current study available from the corresponding author on reasonable request.
